# Pasta with Kiwiberry (*Actinidia arguta*): Effect on Structure, Quality, Consumer Acceptance, and Changes in Bioactivity during Thermal Treatment

**DOI:** 10.3390/foods11162456

**Published:** 2022-08-15

**Authors:** Agata Osoś, Patrycja Jankowska, Agnieszka Drożdżyńska, Maria Barbara Różańska, Róża Biegańska-Marecik, Hanna Maria Baranowska, Millena Ruszkowska, Miroslava Kačániová, Agnieszka Tomkowiak, Marek Kieliszek, Przemysław Łukasz Kowalczewski

**Affiliations:** 1 Students’ Scientific Club of Food Technologists, Poznań University of Life Sciences, 31 Wojska Polskiego St., 60-624 Poznań, Poland; 2 Department of Biotechnology and Food Microbiology, Poznań University of Life Sciences, 48 Wojska Polskiego St., 60-637 Poznań, Poland; 3 Department of Food Technology of Plant Origin, Poznań University of Life Sciences, 31 Wojska Polskiego St., 60-624 Poznań, Poland; 4 Department of Physics and Biophysics, Poznań University of Life Sciences, 38/42 Wojska Polskiego St., 60-637 Poznań, Poland; 5 Faculty of Management and Quality Science, Gdynia Maritime University, 83 Morska St., 81-225 Gdynia, Poland; 6 Institute of Horticulture, Faculty of Horticulture and Landscape Engineering, Slovak University of Agriculture, Tr. A. Hlinku 2, 94976 Nitra, Slovakia; 7 Department of Bioenergy, Food Technology and Microbiology, Institute of Food Technology and Nutrition, University of Rzeszow, 4 Zelwerowicza St., 35-601 Rzeszow, Poland; 8 Department of Genetics and Plant Breeding, Poznań University of Life Sciences, 11 Dojazd St., 60-632 Poznań, Poland; 9 Department of Food Biotechnology and Microbiology, Institute of Food Sciences, Warsaw University of Life Sciences—SGGW, 02-776 Warsaw, Poland

**Keywords:** antioxidant properties, cooking behaviors, functional food, microstructure, molecular water dynamics, pasta enrichment

## Abstract

In this study, kiwiberry lyophilizate (KBL) was incorporated into pasta at different levels (5%, 10%, and 15% *w*/*w*). Kiwiberry fruits’ characteristics (ascorbic acid, carotenoids, phenolic compounds, and antioxidant activity determination) as well as physical (cooking properties, color, microscopic structure determination, texture, and water molecular dynamics analysis by low-field NMR) and chemical analyses (proximate composition phenolic compounds composition and antioxidant activity) of KBL-enriched pasta were investigated. The replacement of semolina with KBL in the production of pasta significantly changed its culinary properties. Results showed that the addition of KBL leads to a reduction in optimal cooking time and cooking weight (47.6% and 37.3%, respectively). Additionally, a significant effect of the KBL incorporation on the color of both fresh and cooked pasta was observed. A significant reduction in the L* value for fresh (27.8%) and cooked (20.2%) pasta was found. The KBL-enriched pasta had a different surface microstructure than the control pasta and reduced firmness (on average 44.7%). Low-field NMR results have confirmed that the ingredients in kiwiberry fruit can bind the water available in fresh pasta. The heat treatment resulted in increasing the availability of phenolic compounds and the antioxidant activity (64.7%) of cooked pasta. Sensory evaluation scores showed that the use of 5–10% of the KBL additive could be successfully accepted by consumers.

## 1. Introduction

Kiwiberry (*Actinidia arguta*), known also as hardy kiwifruit, mini kiwi, baby kiwi, cocktail kiwi, or kiwibes, is the size of a large grape with jelly-like, sweet and sour, green or red flesh and lots of tiny seeds. Unlike typical kiwi fruit, this fruit can be eaten with the skin on, without having to peel them [1]. It is considered a source of many valuable nutrients, including vitamins, polyphenols, carotenoids, and minerals [2]. Published scientific research results indicate the health-promoting properties of these fruits, such as the ability to reduce the risk of cardiovascular diseases and cancer, and help slow the aging process [1]. Among the reported biological activities of kiwiberry fruit are anticancer [3], anti-inflammatory [4], immunomodulating [5], antidiabetic [4,6], and anti-dermatitis [7], as well as bone marrow cell promoting [4,8] and pancreas lipase inhibition properties [9].

Kiwiberries harvested in the phase of bulk maturity can be stored in refrigerated conditions for up to 6 weeks, but only for about 1 week when ripe for consumption. During storage, the content of bioactive compounds is significantly reduced, therefore these fruits require delicate and quick protective treatment or processing [10]. Currently, the main direction of their use is direct consumption or processing into jams, juices, or compotes. To date, however, these fruits were not used to enrich pasta and could become a new, health-promoting ingredient in different types of meals.

Pasta is consumed widely and is considered a staple food in many countries. Its production is easy, and the resulting pasta is cheap and convenient to use, both for dry and sweet dishes [11]. Unlike other grain products, the consumption of pasta does not cause sudden glycemic changes, which is especially important in the case of people suffering from diabetes [12]. The basic raw material used in the production of pasta is wheat semolina, which is hydrated to the appropriate level and then pressed or formed by the use of rolls. Due to its widespread consumption and universal use in nutrition, pasta is often used as a matrix for introducing new, biologically active compounds and nutrients [13,14,15,16,17,18,19,20].

However, the use of additives in the production of pasta may affect its properties, especially its structure and texture, due to the reduction of the share of gluten proteins. Wheat gluten is the most important structural factor of pasta [21]. Reducing the amount of gluten may result in a product that is less compact and more prone to damage, and after cooking such pasta may not maintain its shape [22].

This paper is a report on the effect of replacing semolina with freeze-dried kiwiberries on the quality of the obtained pasta and biological activity. Basic quality indicators, such as cooking weight, cooking loss, optimal cooking time, color, and texture, were assessed, and the change in molecular dynamics of water in fresh and cooked pasta was analyzed using the low-field nuclear magnetic resonance method (LF NMR). Moreover, the content of phenolic compounds and the antioxidant activity of the pasta were assessed before and after the cooking process. A sensory analysis was also carried out to verify whether the new pasta would meet consumer acceptance.

## 2. Materials and Methods

### 2.1. Plant Materials

The semolina from *Triticum durum* was purchased from the mill in Radzyń Podlaski (Poland).

Kiwiberries (*Actinidia arguta*) were obtained from the Frubio Kamil Osoś (Białośliwie, Poland) orchard farm (53°06′49.3″ N 17°06′28.1″ E). They were harvested after achieving harvest maturity (hard fruit, brown seeds) and stored in cold storage until consumption at maturity. The fruits were washed thoroughly, dried, minced with a blender (MaxoMixx, Robert Bosch GmbH, Gerlingen-Schillerhöhe, Germany), and freeze-dried. Then kiwiberry lyophilizate (KBL) was ground, packed, and stored until used.

### 2.2. Kiwiberry Characteristics

The determination of ascorbic acid content in kiwiberries was made in accordance with the method proposed by Biegańska-Marecik et al. [23]. Briefly, the kiwiberries were homogenized with meta-phosphoric acid, shaken (15 min), and centrifuged (4000× *g*, 30 min). The extraction was performed twice and supernatants from both extractions were combined. The samples thus obtained were analyzed with high-performance liquid chromatography (HPLC) using an external standard.

Carotenoids in kiwiberries were extracted with acetone and measured in accordance with the method proposed and described in detail by Szutowska et al. [24], with HPLC. External standards of lutein and β-carotene were used for quantification.

Vallejo et al.’s [25] method was used to extract polyphenols from kiwiberries, and then the obtained polyphenols were determined by HPLC according to the procedure described by Tsao et al. [26].

The total antioxidant activity of the kiwiberries was measured according to Re et al. [27], and the results were expressed as Trolox equivalent per 1 g of fresh weight (f.w.).

### 2.3. Preparation of Pasta

A Häussler Emma (Karl-Heinz Häussler GmbH, Altheim, Germany) pasta extruder was used to produce the pasta analyzed here. Mixes of semolina and KBL in various proportions were hydrated to 33% of water content, with continuous mixing for 20 min, and pressed. Fresh pasta was then dried in a TH-TG-180 (Jeio Tech, Daejeon, Republic of Korea) humidity chamber. The tested pasta formulations contained 5%, 10%, or 15% KBL (semolina was replaced with KBL), and were denoted as K5, K10, and K15, respectively. As the reference, pasta without the addition of KBL was used, denoted as R.

### 2.4. Proximate Composition of Pasta

The protein content was determined according to ISO 20483 [28], the ash content was determined according to AACC Method 08-12.01 [29], and the moisture content was determined according to AACC Method 44-19.01 [30].

### 2.5. Cooking Properties

#### 2.5.1. Optimal Cooking Time

The determination of the optimal cooking time was performed following the procedure proposed by Phongthai et al. [16]. Briefly, Each of the analyzed pasta variants was boiled in water using a ratio of 1:20. A sample was taken out every 15 s and the optimal cooking time was determined as the time needed for the white core, visible in the center of the cross-section of the pasta, to disappear.

#### 2.5.2. Cooking Weight

The cooking weight was determined according to Zardetto et al. [31]. A portion of about 100 g of pasta was weighed and boiled in 1 L of water. After cooking, the sample was drained, dried gently using a paper towel, and weighed after 3 min. Cooking weight was calculated as the ratio of the masses of cooked to raw pasta (g/g).

#### 2.5.3. Cooking Loss

Cooking loss was determined in accordance with the method by Zardetto et al. [31] by weighting the residue obtained after drying (105 °C) the water in which the pasta was cooked during the determination of cooking weight.

### 2.6. Color Measurements

The colors of fresh pasta and cooked pasta were determined using a colorimeter (Chroma Meter CR-410, Konica Minolta Sensing Inc., Tokyo, Japan). The result was presented using a CIE L*a*b* scale, where L* represents lightness, and a* and b* represent redness and yellowness, respectively. Moreover, the results were used to calculate the total color difference (ΔE) according to the formula [16]:

$$\Delta E=\sqrt{\Delta L^{*2}+\Delta a^{*2}+\Delta b^{*2}}$$

### 2.7. Microscopic Structure Determination

A scanning electron microscope (SEM) (Zeiss EVO 40, Carl Zeiss AG, Oberkochen, Germany) was used for the study of the microstructure of dry pasta. Preparation of the samples included cross-sectioning of the dried pasta, mounting the sections onto aluminum discs using a silver paste, and coating with gold using a JEE-400 vacuum dryer (Jeol, Tokyo, Japan).

### 2.8. Texture Analysis

Textural properties of cooked pasta were determined using a TA.XTplus texture analyzer (Stable Micro System, Godalming, UK) equipped with a plexiglass straight probe, according to the previously described method [14]. The dimensions of the pasta samples were 2 mm (height) × 10 mm (width) × 100 mm (length). Each sample was measured in 15 replicates. Pasta samples were placed in a single layer on the measurement plate so that each adhered to the others. The test was performed to ensure the complete cutting of the pasta. Firmness and work of shear were measured.

### 2.9. ^1^H NMR Relaxometry

Water molecular dynamics analysis was performed for fresh (immediately after the pressing process) and cooked pasta.

The samples were placed in test tubes (8 mm diameter) and sealed using Parafilm^®^. A pulse NMR spectrometer operating at 15 MHz (Ellab, Poznań, Poland) was used to measure the spin-lattice (T_1_) and spin-spin (T_2_) relaxation times. T_1_ relaxation times were measured using the inversion-recovery (π–*TI*–π/2) impulse sequence. Distances between RF pulses (*TI*) were changed within the range from 0.4 to 40 ms for fresh pasta samples and from 20 to 100 ms for cooked pasta samples. The repetition times were 15 s for both kinds of pasta. Each time, 32 FID signals with 119 points from each FID signal were collected. CracSpin program was used to calculate the values of spin-lattice relaxation time with the assistance of the spin grouping approach [32]. The following formula describes time changes in the current value of the FID signal amplitude in the employed frequency of impulses:

$$M_{z}\left(TI\right)=\;M_{0}\left(1-2e^{\frac{-TI}{T_{1}}}\right)$$

where: M_z_ (*TI*) is the actual magnetization value, and M_0_ is the equilibrium magnetization value.

Both types of samples manifested mono-exponential magnetization recovery. Measurements of the T_2_ spin-spin relaxation times were made using the pulse train of the Carr-Purcell-Meiboom-Gill spin echoes (π/2–*TE*/2–(π)_N_). The distance between π (*TE*) impulses was 0.2 ms for fresh pasta and 1 ms for cooked pasta samples. The repetition time was 15 s. The number of spin echoes (N) amounted to 100. Ten accumulation signals were employed. To calculate the spin-spin relaxation time values, the following formula was used [33]:

$$M_{x,y}\left(\mathrm{TE}\right)=M_{0}\overset{n}{\underset{i=1}{\sum }}p_{1}e^{\frac{-\mathrm{TE}}{T_{2i}}}$$

where: M_x,y_(TE) is the echo amplitude; M_0_ is the equilibrium amplitude; and p is the fraction of protons relaxing with the T_2i_ spin-spin time.

A two-exponential spin echo delay was found for both types of samples. This means that protons relaxed with two separated relaxation times.

### 2.10. Changes of Pasta Antioxidant Activity during Thermal Processing

#### 2.10.1. Extraction Process of Antioxidants

Samples of dry and cooked pasta samples were freeze-dried before methanolic (80% methanol solution in water) extraction in the ratio of pasta and solvent 1:5. Then extraction was performed for 45 min, the samples were centrifuged (12000× *g*, 10 min), and supernatants were decanted, filtered and stored at −20 °C prior to the analyzes.

#### 2.10.2. Total Antioxidant Activity and Total Phenolic Content

ABTS radical method was used to determine the antioxidant activity [27] and the results were expressed as Trolox equivalent antioxidant capacity (TEAC) per 1 g of d.m. The Folin-Ciocalteu method was used to determine the total phenolic compounds (TPC) [34], which was expressed as ferulic acid equivalent (FAE) per 1 g of d.m.

#### 2.10.3. Polyphenols Profile Composition

Analyzes of phenolic compound contents in dry and cooked pasta samples were performed using liquid chromatography (Agilent 1260 Infinity II Agilent Technologies, Inc., Santa Clara, CA, USA) according to the method described in detail previously [35]. The results were obtained based on the signals at the following detector wavelengths: 320 nm for caffeic, *p*-coumaric, sinapic, and ferulic acids; vitexin at 340 nm for vitexin; 360 nm for rutin, kaempferol, and quercetin; 255 nm for chlorogenic acid. Quantitation was based on peak areas. OpenLab CDS was used for data analysis.

### 2.11. Consumer Acceptance

Forty untrained testers, aged between 20–40, were asked to evaluate the appearance, color, flavor, taste, texture, and overall rating of pasta samples. The 9-point hedonic line scale (from 1—dislike very much to 9—like very much) was used to rate consumer acceptance of cooked pasta [36].

### 2.12. Statistical Analysis

All tests were carried out in triplicate for two parallel tests (*n* = 6) unless stated otherwise. One-way analysis of variance (ANOVA) was performed with Statistica 13 software (Dell Software Inc., Round Rock, TX, USA). Statistically homogeneous subsets were identified with a post-hoc Tukey HSD multiple comparison test at α = 0.05.

## 3. Results and Discussion

### 3.1. Kiwiberry Properties

Kiwi fruit is a rich source of vitamins, among which vitamin C is especially important. The content of this vitamin varies between species but growing conditions also affect its level [37,38,39,40,41]. In the kiwiberries used in the further part of the research, high content of vitamin C was also determined at the level of 55.6 mg in 100 g of fruit (Table 1), so its level is comparable to other studies. Among the various pigments found in *Actinidia* fruits are carotenoids, chlorophylls, and anthocyanins. In addition, among carotenoids, lutein and β-carotene make up the vast majority [38], consistent with the results obtained in our studies. The presence of other pigments in fruits, such as zeaxanthin or violaxanthin, is also confirmed by literature data at levels similar to those indicated here [42,43]. Moreover, kiwiberries are significant sources of phenols, widely recognized as powerful antioxidants. It is worth mentioning, that the content of these secondary metabolites depends on the growing conditions of plants, as well as the influence of stress factors, both abiotic and biotic [44,45,46]. The more stress factors acted during the growth of the plant, the greater the content of phenolic compounds. As pointed out by Park et al. [47], the main group of phenols in *A. arguta* fruits are flavonoids, tannins, and flavanols. Other published studies indicate that these fruits contain significant amounts of gallic acid, chlorogenic acid, tannic acid, 2,4-dihydroxybenzoic acid, caffeic acid, (+)-catechin, (−)-epicatechin, rutin, and quercetin [48,49]. Our research results are in line with the literature data and confirm the high content of phenolic compounds in kiwiberries, which determines high antioxidant activity, determined by the ABTS method, also described in the literature [2].

### 3.2. Proximate Composition and Pasta Cooking Properties

As expected, the replacement of semolina with KBL resulted in a decrease in the protein content of the pasta and an increase in the content of ash (Table 2). On the other hand, there was no effect on the moisture content of pasta, which was dried under unchanged conditions each time.

The most important indicators of the quality of pasta are its culinary properties, including the ability of the pasta to absorb water during cooking (cooking weight), but also the amount of dry matter lost (cooking loss). As indicated by the published data [50,51,52,53], the use of additives in the production of pasta can significantly change these properties, reducing the quality of pasta. Due to the reduction of the protein content, and consequently, of gluten, which is an important structural factor of pasta [12], a reduction in the optimal cooking time was observed along with the increase in KBL share in the pasta recipe (6.30 for R vs. 3.30 min for K15), as well as a significant reduction in cooking weights (2.17 vs. 1.36 g/g, respectively). The cooking time can also significantly affect the cooking loss [54], therefore, to mark these, the pasta was cooked at the optimal time. Increasing the cooking loss, due to the enrichment of pasta with KBL, may also be a result of the weakening of the gluten matrix, which resulted in weaker binding of starch granules in the structure of the pasta amylose leaching and solubilization of some salt-soluble proteins into the water during cooking [55]. Other authors also observed an increase in cooking loss in pasta enriched with new ingredients [18,50,56].

### 3.3. Color Parameters

**Figure 1:**
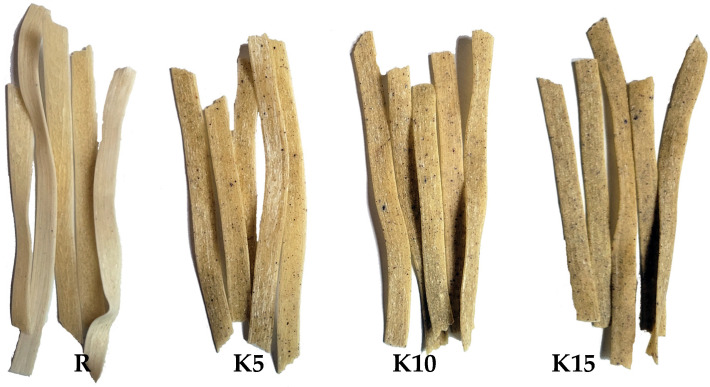
The overall appearance of obtained pasta. R—reference pasta without the addition of kiwiberry lyophilizate; K5, K10, and K15—pasta contained 5%, 10%, and 15% kiwiberry lyophilizate.

The use of KBL in the pasta recipe changed the appearance of the pasta, which was already visible in dry pasta (Figure 1). The greater the proportion of KBL in the pasta, the darker its color and the more dark motes in the pasta, due to the numerous small seeds present in KBL.

The color of the pasta is extremely important because it can be assessed by consumers when deciding to buy a specific product in a store [57]. Literature data indicate that the color change is not always accepted by consumers, and new products, despite their health-promoting properties, may be rejected [58]. Therefore, the color of fresh pasta, immediately after the pressing process, was analyzed, as well as the color of cooked pasta, ready to eat (Table 3). A significant reduction in the L* value, corresponding to the lightness of the pasta, was noted due to the addition of KBL. However, these changes were smaller in cooked than in fresh pasta. Reducing the lightness of the pasta can be seen as a negative aspect of quality as consumers usually expect pasta with a bright yellow color [59]. The decrease in lightness may be caused both the KBL used itself, which was dark brown, also by the non-enzymatic browning of the reducing sugars and the oxidation of numerous carotenoid pigments in fruit and vegetable products [60,61], also present in KBL (see Table 1). With increasing amounts of KBL the green/red (a*) and blue/yellow (b*) color balances were shifted towards red and yellow, respectively. Similar dependency was observed for pasta enriched with other plant-based ingredients reported in another study [51,62,63]. Smaller differences in the color components of KBL pasta compared to R after the cooking process can be explained by the higher water content in the cooked pasta, but also by the rinsing out and degradation of pigments during cooking [64,65]. The value of ΔE indicates a significant effect of the addition of KBL on the color of both fresh and cooked pasta. The color difference of the analyzed pasta, calculated based on the L*a*b* color components, was from 10.34 to 22.10 in fresh pasta, and from 7.02 to 17.27 in cooked pasta. Mokrzycki and Tatol [66] pointed out that ΔE > 2 indicates a significant change in the color of the food product, and the color differences may also be noticed by an inexperienced observer.

### 3.4. Texture and Microstructure

Two texture parameters (firmness and total shear work) of pasta with and without the addition of KBL were analyzed. Dziki et al. [67] showed that pasta firmness decreased with cooking time. All pasta was therefore prepared for texture analysis by cooking at the optimal cooking time (see Section 3.2). It has been shown that KBL-enriched pasta is characterized by lower firmness, and less work is required to cut the pasta completely (Table 4). The observed changes were greater as the greater proportion of the semolina was replaced with KBL. The firmness of pasta depends on the content of gluten and amylopectin. The increase in gluten and amylose content improves the firmness of the finished product [68,69], but it is well known that KBL does not contain either gluten or amylose. As more KBL was added, the semolina gluten was effectively diluted, resulting in a reduction in pasta firmness. Additionally, published data [70] indicate that the greater the firmness of the pasta, the smaller the loss of weight during its cooking. The lower firmness may therefore explain the increased losses (see Section 3.2).

**Figure 2:**
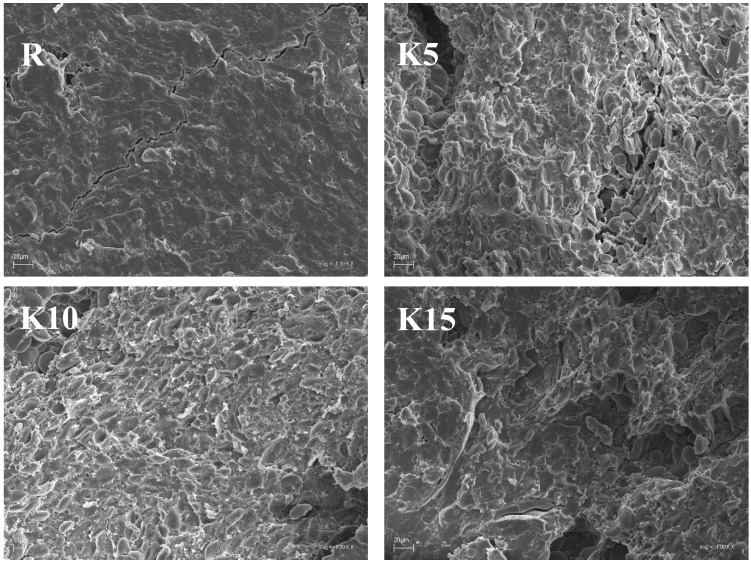
Scanning electron microphotographs of pasta. R—reference pasta without the addition of kiwiberry lyophilizate; K5, K10, and K15—pasta contained 5%, 10%, and 15% kiwiberry lyophilizate.

SEM studies explain the observed differences in texture and culinary properties of the studied kinds of pasta. The KBL-enriched pasta had a different surface microstructure than the control pasta (Figure 2). The microstructure of pasta depends on many factors; however, according to Lucisano et al. [71], the most important factor determining the structure of pasta is the interaction between the starch and the protein fraction. Based on the observation of the dry pasta microstructure Resmini and Pagani [72] showed, that the gluten network is arranged regularly around the starch granules, creating a homogeneous structure, which is also observed in the case of the reference pasta (R) in this study (Figure 2). The use of KBL in the production of pasta reduced the content of gluten, which resulted in the obtaining of pasta with a looser structure. The starch granules are also visible which are no longer so tightly bound to the gluten network. The presence of pores in the structure of pasta may be related to the pressing process, but their quantity and size are determined by the rheological properties of the dough resulting from its composition. Reducing the gluten content of KBL-enriched pasta facilitates the swelling of starch during pasta cooking, reducing its firm texture and increasing the viscosity of the pasta [72]. It also explains the observed increase in cooking losses (see Section 3.2), as the part of the starch that is not bound to the gluten network can pass into the boiling water.

### 3.5. Water Behavior

Low-field NMR is a widely used method to describe the molecular properties of water in various biological samples, including food products [73,74,75,76,77]. The water in fresh pasta can be associated with the ingredients in the flour (starch, gluten), but also with added ingredients such as the sugars (in their various states) present in the kiwifruit. It was noticed that the bigger part of the semolina was replaced with KBL, and the lower T_1_ time values were recorded (Table 5). This means that the ingredients present in the fruit bound the water available in fresh pasta. During cooking, the pasta absorbs water, and as a consequence, there are numerous interactions between water and macromolecules, which additionally changes the characteristics of molecular dynamics [78]. In this case, significantly lower T_1_ values of KBL-enriched pasta were also observed compared to R. The obtained results indicate that the water present in both fresh and cooked pasta remains mainly outside the starch granules [79], interacting preferentially with the sugars present in the dough derived from KBL. Mutual competition between saccharides and starch for water was also confirmed by other researchers [80].

The analysis of the results of the T_21_ time measurements (Table 5) showed that the values of this time increased significantly (in each case about 10 times) after the pasta was cooked, which may be due to the additional water uptake by the starch granules. This may be related to the increase in the free volume in which the starch granules can move freely, and to the proton population, possibly related to water [81]. Water–starch and water–gluten interactions, which compete with each other, may cause differences between the obtained T_2_ time values for water instead of labile protons (in -OH, -NH, and -SH functional groups) from starch and gluten [82]. Native wheat starch granules are highly hydrophobic; however, during heat treatment (cooking), the granules absorb water, significantly swelling [83], which explains the increase in T_21_. The presence of sugars, including sucrose, glucose, and fructose, derived from KBL changed the interaction between water and wheat starch. In such a case, water exhibits stronger interactions with other saccharides than with starch, which is manifested by an extension of the T_22_ time in pasta with KBL, both fresh and after the cooking process. At the same time, the amount of water that the starch granules retain is smaller, hence the observed lower values of the T_21_ time in KBL-enriched paste than in R.

### 3.6. Changes of Antioxidant Activity and Phenolic Compounds Composition during Thermal Processing

Semolina, used in the production makes it possible to obtain good-quality pasta, but low in phenolic compounds with antioxidant properties [84,85]. As mentioned earlier (see Section 3.1), kiwiberries are a rich source of antioxidant compounds, including polyphenolic compounds. Therefore, the enrichment of pasta in KBL can significantly increase the biological activity of the obtained pasta. Table 6 presents the results of the total polyphenol content, antioxidant activity, as well as the detailed composition of phenolic compounds in the dry and cooked pasta to check how the heat treatment affects the antioxidant activity of the new pasta. The observed increase in TPC and TEAC values in KBL-based pasta was unexpected, and the observed changes were the greater the more KBL was added to the pasta. The formation of new compounds as a result of non-enzymatic browning, i.e., polymeric melanoidins in Maillard reactions [86,87], during the drying of fresh pasta probably also contributed to the increase in antioxidant activity. Moreover, in addition to free phenolic acids and polyphenols (see Table 1), kiwiberries and semolina contain bound phenolic compounds that are linked to cell wall polysaccharides by ester bonds and may be released during cooking [88]. The highest content was recorded for chlorogenic acid, rutin, and vitexin, and thermal treatment significantly increased the availability of these compounds, causing an increase in antioxidant activity. Other published studies have also shown that heat treatment can increase antioxidant activity and release bound phenolic compounds [89,90,91].

### 3.7. Consumer Study

**Figure 3:**
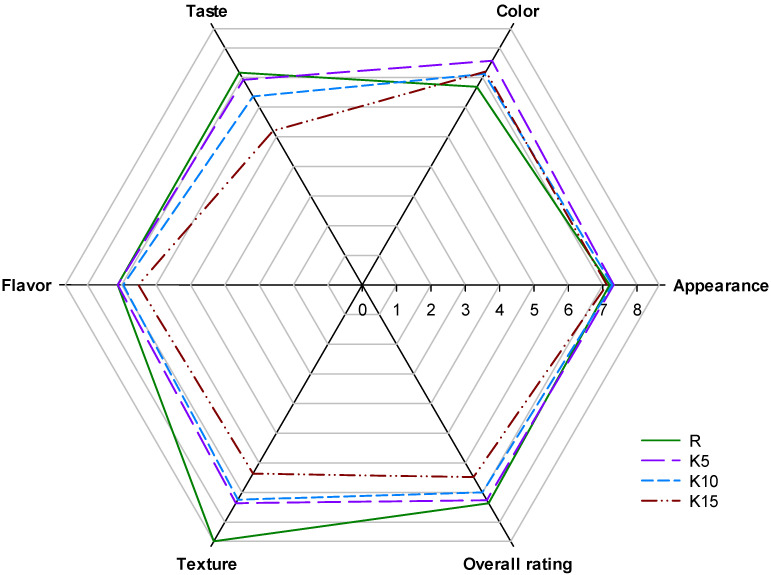
Results of consumer study of cooked pasta. R—reference pasta without the addition of kiwiberry lyophilizate; K5, K10, and K15—pasta containing 5%, 10%, and 15% kiwiberry lyophilizate.

On the one hand, the use of new ingredients for the production of pasta may improve its market value or give it new health-promoting properties, but on the other hand, they may affect the properties of the final product. To successfully incorporate a new pasta on the market, it must therefore have appropriate sensory characteristics that are acceptable to consumers [92]. The changes observed in previous analyzes, both macroscopic and microscopic, may therefore make it difficult to use KBL as an ingredient in the production of pasta. There were no differences in the assessment of the external appearance of pasta, both enriched with and without KBL (Figure 3 and provided in the detail in Table S1). The color of KBL-enriched pasta was also assessed as surprisingly good, and the values of the grades were significantly higher than those of R. This could be explained by the fact that the color resembled commercially available whole meal paste, which is widely recognized as being healthier. Unfortunately, KBL had a negative impact on the other three assessed parameters, i.e., taste, smell, and texture. In the case of the first two parameters, the use of a 5% additive did not significantly affect the perception of pasta by consumers, compared to the traditional product. However, the biggest differences were observed in the case of texture. Due to changes in consistency, the texture of enriched pasta was rated lower than pasta without KBL. Interestingly, the K5 pasta obtained a general desirability rating identical to the R pasta. The K10 rating also did not differ statistically significantly. A further increase in the KBL share in pasta resulted in a reduction in the grades awarded. It can therefore be assumed that consumers can successfully accept the use of 5–10% of the additive.

## 4. Conclusions

Eliminating oxidative stress by supplying antioxidant ingredients to the diet can reduce the risk of numerous diseases. Therefore, the use of new ingredients in food production is the subject of research by many scientific centers around the world. Based on the research conducted here, it has been shown that kiwiberry fruit can be successfully used to enrich pasta with new, valuable health-promoting components. Importantly, the heat treatment further enhances the antioxidant capacity, most likely through the release of bound phenolic compounds. The addition of KBL also caused numerous changes in the properties of the pasta, observed both at the macroscopic and micro levels. However, based on the consumer analysis, it was found that pasta with KBL is positively assessed by the respondents, thus it is possible to use this valuable source of bioactive compounds, to create new pasta formulations.

## Figures and Tables

**Table 1:** Characteristics of kiwiberries used in this study (*n* = 6).

Parameter	Amount
Vitamin C [mg/100 g f.m.]	54.658 ± 3.796
Carotenoids content (μg/100 g f.m.):	
Neoxanthin	3.412 ± 0.407
Violaxanthin	4.780 ± 0.499
Anteraxanthin	4.339 ± 0.442
Lutein	19.003 ± 2.122
Zeaxanthin	1.618 ± 0.223
β-carotene	19.447 ± 2.601
Not identified	1.468 ± 0.358
Individual polyphenolic compound (mg/100 g f.m.):	
Hydroxybenzoic acid	3.311 ± 0.131
Catechin	2.056 ± 0.150
Procyanidin B	3.742 ± 0.323
Epicatechin	2.064 ± 0.142
Procyanidins	4.525 ± 0.241
Neochlorogenic acid	10.537 ± 0.795
*p*-Coumaric acid	2.656 ± 0.293
Chlorogenic acid	11.138 ± 0.881
Crypto-chlorogenic acid	3.517 ± 0.189
Caffeic acid	0.475 ± 0.048
Rutin	12.402 ± 2.226
Quercetin	4.231 ± 0.824
Kaempferol	3.951 ± 0.271
Antioxidant activity * [μmol TEAC/1 g f.m.]	84.70 ± 3.36

TEAC: Trolox equivalent antioxidant capacity. * According to the ABTS method.

**Table 2:** Proximate composition and cooking behavior of analyzes pasta.

Parameter	R	K5	K10	K15
Protein content (%)	11.0 ± 0.2 ^a^	10.8 ± 0.1 ^ab^	10.1 ± 0.2 ^b^	9.9 ± 0.5 ^b^
Ash content (%)	0.763 ± 0.005 ^d^	0.828 ± 0.006 ^c^	0.916 ± 0.007 ^b^	1.025 ± 0.015 ^a^
Moisture content (%)	6.77 ± 0.11 ^a^	6.74 ± 0.21 ^a^	6.62 ± 0.17 ^a^	6.83 ± 0.22 ^a^
Optimal cooking time (min)	6.30 ± 0.05 ^a^	4.30 ± 0.10 ^b^	4.00 ± 0.02 ^c^	3.30 ± 0.03 ^d^
Cooking weight (g/g)	2.17 ± 0.21 ^a^	2.07 ± 0.11 ^a^	1.77 ± 0.06 ^b^	1.36 ± 0.07 ^c^
Cooking loss (%)	6.42 ± 0.83 ^b^	7.22 ± 1.02 ^b^	10.04 ± 2.14 ^ab^	14.11 ± 1.72 ^a^

Mean values denoted by different letters (a–d) in rows differ statistically significantly (*p* < 0.05). R—reference pasta without the addition of kiwiberry lyophilizate, K5, K10, and K15—pasta containing 5%, 10%, and 15% kiwiberry lyophilizate.

**Table 3:** Color parameters of pasta enriched with kiwifruit.

Parameter	Fresh Pasta				Cooked Pasta			
	R	K5	K10	K15	R	K5	K10	K15
L*	77.6 ± 0.4 ^a^	67.9 ± 1.2 ^b^	60.2 ± 0.5 ^c^	56.0 ± 0.2 ^d^	74.2 ± 0.2 ^A^	68.7 ± 0.2 ^B^	64.3 ± 0.2 ^C^	59.2 ± 0.3 ^D^
a*	0.50 ± 0.12 ^a^	−2.58 ± 0.27 ^b^	−2.29 ± 0.06 ^c^	−2.34 ± 0.10 ^bc^	−2.94 ± 0.07 ^D^	−2.05 ± 0.07 ^C^	−0.98 ± 0.08 ^B^	1.05 ± 0.02 ^A^
b*	31.8 ± 0.3 ^b^	32.9 ± 1.6 ^a^	33.6 ± 0.3 ^ab^	38.0 ± 0.3 ^c^	13.6 ± 0.2 ^C^	19.3 ± 0.6 ^B^	22.3 ± 0.9 ^A^	21.2 ± 1.6 ^AB^
ΔE	-	10.34	17.67	22.10	-	7.92	13.31	17.27

Mean values denoted by different letters in rows (a–d for fresh and A–D for cooked pasta) differ statistically significantly (*p* < 0.05). R—reference pasta without the addition of kiwiberry lyophilizate; K5, K10, and K15—pasta contained 5%, 10%, and 15% kiwiberry lyophilizate. L*—lightness, a*—redness, b*—yellowness, ΔE—the total color difference.

**Table 4:** Texture parameters of cooked pasta.

Parameter	R	K5	K10	K15
Firmness [N]	2.82 ± 0.41 ^a^	1.72 ± 0.44 ^b^	1.57 ± 0.21 ^bc^	1.39 ± 0.13 ^c^
Total work of shear [N/mm × s]	0.143 ± 0.027 ^a^	0.101 ± 0.029 ^ab^	0.097 ± 0.012 ^bc^	0.079 ± 0.010 ^c^

Mean values denoted by different letters (a–c) in rows differ statistically significantly (*p* < 0.05). R—reference pasta without the addition of kiwiberry lyophilizate; K5, K10, and K15—pasta contained 5%, 10%, and 15% kiwiberry lyophilizate.

**Table 5:** LF NMR relaxometry results of fresh and cooked pasta.

Parameter	Fresh Pasta				Cooked Pasta			
	R	K5	K10	K15	R	K5	K10	K15
T_1_ (ms)	56.7 ± 0.3 ^a^	49.9 ± 1.7 ^b^	39.3 ± 3.9 ^c^	34.6 ± 2.8 ^d^	264.0 ± 0.8 ^A^	254.1 ± 1.2 ^B^	154.1 ± 1.3 ^C^	131.5 ± 0.8 ^D^
T_21_ (ms)	1.21 ± 0.09 ^a^	0.83 ± 0.02 ^b^	0.68 ± 0.04 ^c^	0.60 ± 0.04 ^c^	18.1 ± 0.5 ^A^	13.0 ± 0.3 ^B^	9.8 ± 0.7 ^C^	7.7 ± 0.2 ^D^
T_22_ (ms)	2.09 ± 0.05 ^c^	2.21 ± 0.05 ^b^	2.32 ± 0.03 ^a^	2.38 ± 0.04 ^a^	66.1 ± 1.1 ^D^	76.7 ± 0.9 ^C^	79.0 ± 0.2 ^B^	81.2 ± 0.6 ^A^

Mean values denoted by different letters in rows (a–d for fresh and A–D for cooked pasta) differ statistically significantly (*p* < 0.05). R—reference pasta without the addition of kiwiberry lyophilizate; K5, K10, and K15—pasta contained 5%, 10%, and 15% kiwiberry lyophilizate.

**Table 6:** Results of antioxidant activity and phenolic compounds composition after cooking per 1 g of dry matter.

Parameter	Dry Pasta				Cooked Pasta			
	R	K5	K10	K15	R	K5	K10	K15
Antioxidant activity and total phenolic compound content								
TPC (mg FAE/g)	0.31 ± 0.03 ^d^	0.57 ± 0.01 ^c^	0.72 ± 0.04 ^b^	1.03 ± 0.05 ^a^	0.61 ± 0.02 ^D^	1.16 ± 0.02 ^C^	1.93 ± 0.03 ^B^	2.41 ± 0.06 ^A^
TEAC (mmol/g)	12.25 ± 0.26 ^b^	13.31 ± 0.74 ^b^	14.23 ± 0.42 ^ab^	15.86 ± 2.50 ^a^	10.95 ± 0.06 ^C^	14.86 ± 0.73 ^B^	16.35 ± 0.75 ^A^	18.04 ± 1.96 ^A^
Phenolic compounds composition								
Chlorogenic acid (μg/g)	98.9 ± 28.0 ^c^	1791 ± 41 ^b^	3856 ± 83 ^ab^	8133 ± 443 ^a^	70.9 ± 29.4 ^D^	2348 ± 42 ^C^	5700 ± 749 ^B^	10870 ± 948 ^A^
Quercetin (μg/g)	N/D	765.6 ± 13.5 ^c^	987.8 ± 31.6 ^b^	1349 ± 82 ^a^	N/D	1099 ± 19 ^C^	1580 ± 51 ^B^	2076 ± 73 ^A^
Rutin (μg/g)	164.2 ± 6.1 ^d^	2496 ± 43 ^c^	5083 ± 80 ^b^	9888 ± 199 ^a^	432.9 ± 429.8 ^D^	2440 ± 439 ^C^	5456 ± 16 ^B^	9901 ± 464 ^A^
Kaempferol (μg/g)	N/D	N/D	N/D	N/D	N/D	N/D	N/D	438.30 ± 37.22
Caffeic acid (μg/g)	N/D	355.7 ± 12.3 ^c^	610.4 ± 52.5 ^b^	1271 ± 188 ^a^	N/D	784.7 ± 28.4 ^C^	1510 ± 158 ^B^	2408 ± 98 ^A^
*p*-Coumaric acid (μg/g)	582.0 ± 37.4 ^c^	1031 ± 11 ^b^	1212 ± 136 ^b^	1660 ± 178 ^a^	477.1 ± 77.8 ^C^	1279 ± 146 ^B^	1608 ± 373 ^AB^	2134 ± 207 ^A^
Vitexin (μg/g)	2748 ± 27 ^b^	2825 ± 78 ^b^	3360 ± 69 ^a^	3969 ± 104 ^a^	2409 ± 100 ^C^	3077 ± 230 ^B^	3340 ± 64 ^B^	3758 ± 182 ^A^
Ferulic acid (μg/g)	1762 ± 27 ^c^	1848 ± 71 ^bc^	1987 ± 11 ^b^	2752 ± 57 ^a^	1606 ± 16 ^C^	1957 ± 116 ^B^	2043 ± 193 ^B^	2714 ± 166 ^A^
Sinapic acid (μg/g)	82.7 ± 1.5 ^c^	223.9 ± 5.9 ^b^	237.1 ± 13.1 ^b^	343.0 ± 24.8 ^a^	89.5 ± 4.8 ^C^	261.1 ± 24.3 ^B^	275.6 ± 29.9 ^B^	357.7 ± 23.8 ^A^

Mean values denoted by different letters in rows (a–d for fresh and A–D for cooked pasta) differ statistically significantly (*p* < 0.05). N/D—not detected. R—reference pasta without the addition of kiwiberry lyophilizate; K5, K10, and K15—pasta containing 5%, 10%, and 15% kiwiberry lyophilizate.

## Data Availability

All data generated or analyzed during this study are included in this published article and its supplementary materials.

## Supplementary Materials

The following supporting information can be downloaded at: https://www.mdpi.com/article/10.3390/foods11162456/s1, Table S1: Results of consumer rating.
