# Supplementary material for: Pasta with Kiwiberry (Actinidia arguta): Effect on Structure, Quality, Consumer Acceptance, and Changes in Bioactivity during Thermal Treatment
Source: Foods. 2022 Aug 15;11(16):2456. doi: 10.3390/foods11162456 (PMC9407217; doi:10.3390/foods11162456)
Supplement: Supplementary file 1 [file foods-11-02456-s001.zip › foods-1842827-supplementary.pdf]

foods

MDPI

Article

Pasta with Kiwiberry (*Actinidia arguta*): Effect on Structure, Quality, Consumer Acceptance, and Changes in Bioactivity during Thermal Treatment

Agata Osoś [1], Patrycja Jankowska [1], Agnieszka Drożdżyńska [2], Maria Barbara Różańska [3], Róża Biegańska-Marecik [3], Hanna Maria Baranowska [4], Millena Ruszkowska [5], Miroslava Kačániová [6,7], Agnieszka Tomkowiak [8], Marek Kieliszek [9] and Przemysław Łukasz Kowalczewski [3,*]

1 Students' Scientific Club of Food Technologists, Poznań University of Life Sciences, 31 Wojska Polskiego St., 60-624 Poznań, Poland
2 Department of Biotechnology and Food Microbiology, Poznań University of Life Sciences, 48 Wojska Polskiego St., 60-637 Poznań, Poland
3 Department of Food Technology of Plant Origin, Poznań University of Life Sciences, 31 Wojska Polskiego St., 60-624 Poznań, Poland
4 Department of Physics and Biophysics, Poznań University of Life Sciences, 38/42 Wojska Polskiego St., 60-637 Poznań, Poland
5 Faculty of Management and Quality Science, Gdynia Maritime University, 83 Morska St., 81-225 Gdynia, Poland
6 Institute of Horticulture, Faculty of Horticulture and Landscape Engineering, Slovak University of Agriculture, Tr. A. Hlinku 2, 94976 Nitra, Slovakia
7 Department of Bioenergy, Food Technology and Microbiology, Institute of Food Technology and Nutrition, University of Rzeszow, 4 Zelwerowicza St, 35-601 Rzeszow, Poland
8 Department of Genetics and Plant Breeding, Poznań University of Life Sciences, 11 Dojazd St., 60-632 Poznań, Poland
9 Department of Food Biotechnology and Microbiology, Institute of Food Sciences, Warsaw University of Life Sciences—SGGW, 02-776 Warsaw, Poland
* Correspondence: przemyslaw.kowalczewski@up.poznan.pl

Table S1. Results of consumer rating.

Parameter	R	K5	K10	K15
Appearance	7.20 ± 0.31 [a]	7.32 ± 0.29 [a]	7.28 ± 0.70 [a]	7.12 ± 0.83 [a]
Color	6.68 ± 0.23 [b]	7.56 ± 0.16 [a]	7.12 ± 0.33 [a]	7.20 ± 0.71 [ab]
Taste	7.16 ± 0.14 [a]	6.92 ± 0.55 [ab]	6.36 ± 0.91 [bc]	5.20 ± 1.12 [c]
Flavor	7.12 ± 0.19 [a]	7.12 ± 0.32 [ab]	6.96 ± 0.55 [b]	6.52 ± 0.68 [b]
Texture	8.64 ± 0.26 [a]	7.36 ± 0.34 [b]	7.24 ± 0.48 [b]	6.36 ± 0.44 [c]
Overall rating	7.36 ± 0.21 [a]	7.26 ± 0.22 [a]	6.99 ± 0.39 [b]	6.48 ± 0.34 [b]

Means in the same row followed by different letters indicate significant differences ($p < 0.05$). R - reference pasta without the addition of kiwiberry lyophilizate, K5, K10, and K15 - pasta contained 5%, 10%, and 15% kiwiberry lyophilizate.
